# Novel Case Presentation of Abulia After Lone Star Tick Bite As Evidenced by Raised Titers of Alpha-Gal Specific IgM Immunoglobulin and a Possibility of Alpha-Gal Driven Hypothalamic Dysfunction As the Pathomechanism

**DOI:** 10.7759/cureus.24551

**Published:** 2022-04-28

**Authors:** Bob Daripa, Scott Lucchese

**Affiliations:** 1 Medicine, Grant Medical College and Sir Jamsetjee Jeejeebhoy (JJ) Group of Government Hospitals, Mumbai, IND; 2 Internal Medicine/Neurology, Singapore General Hospital, Singapore, SGP; 3 Neurology, University Hospital, Missouri University Health Care, Columbia, USA; 4 Neurology, University of Arkansas for Medical Sciences, Little Rock, USA; 5 Neurology, University of Missouri School of Medicine, Columbia, USA

**Keywords:** hypothalamus, hypocretins, auto-immune molecular mimicry, α-gal syndrome, lone star tick, α-gal, 3-galactose, galactose-α-1, abulia

## Abstract

Galactose-α-1,3-galactose, an oligosaccharide epitope better acknowledged as α-Gal, is present in non-primate mammal meat, tick bites, microorganisms, and vaccines as a glycoprotein or glycolipid moiety. This can manifest hyperimmune reactions as it enters the human body, known as α-Gal syndrome (AGS). AGS and Guillain-Barré syndrome share cognate immunogenic pathomechanism via conquering immune tolerance further speculating galactose navigated neurological sequel. Unusual symptomatic presentation of abulia in our case, with incidental finding of high titers of α-Gal specific IgE immunoglobulin further supported by temporal resolution of symptoms on abstinence of meat products, raises a high degree of suspicion of neuro-psychiatric manifestation in sensitized α-Gal patients. The pathomechanism is blurry, and an absence of an objective diagnostic tool makes the neurological diagnosis challenging. α-Gal driven immune-related hypothalamic dysfunction could be a possibility that needs further exploration and is a topic of research.

## Introduction

In 2008, cetuximab, a metastatic colorectal cancer drug, reported anaphylactic reactions in confined parts of the United States (particularly southeastern United States). Later, specific immunoglobulin were identified in these patients in response to galactose-α-1,3 galactose (an oligosaccharide epitope commonly known as α-Gal) [1]. A year later, related reactions and laboratory findings were reported after red meat consumption, but it was a delayed onset (3-6 hours) [2] incident perhaps attributed to the hindered entry of α-Gal linked chylomicrons into circulation through the thoracic duct [1]. Surprisingly, similar symptomatology was published from different parts of the world like Sweden, Australia, and USA (south, eastern, and central states viz. Tennessee, North Carolina, Arkansas, Virginia, and Southern Missouri) [1,2] after consuming mammalian food products (namely beef, pork, lamb, sometimes milk and cheese, but not in chicken, turkey, and fish), particularly in those individuals who previously tolerated red meat pleasantly [1].

A decade later, Commins et al. found a strong association between a particular tick bite (lone star tick - Amblyomma americanum; identified by a white dot on the dorsum of females) and raised immunoglobulin levels against galactose-α-1,3 galactose (α-Gal) [1]. As high as 20% subset of the population in the southeast regions of the United States have already been sensitized to its bite and eventually to α-Gal present in these tick’s immunogenic saliva [1,2]. Even chronic consumption of α-Gal glycosylated red meat, particularly the glycolipid component, exposes humans to α-Gal where there is a repetitive liberation of inflammatory products along with IgE antibodies from mast cells without symptoms [3]. Perhaps these individuals may be considered asymptomatic α-Gal sensitized subjects [1].

On consuming red meat, after a few weeks, these lone star tick bit asymptomatic α-Gal sensitized individuals experience anaphylactic reactions. The possible pathomechanism could be tick saliva-induced IgE immunoglobulin cross-reacting with oligosaccharide α-Gal sugar epitope found in previously consumed non-catarrhine meat products resulting in above hyperimmune reactions [2]. This riposte releases an enormous IgE immunoglobulin precipitating delayed type-1 anaphylactoid reaction [1]. Such sensitized individuals could possibly manifest into unusual neuropsychiatric behavior, as seen in our case where raised titers of α-Gal specific IgE immunoglobulin were the only significant laboratory finding. The related pathomechanism is not clear, although the possibility of auto-immune molecular mimicry affecting hypothalamic nuclei cannot be ruled out.

## Case presentation

A 65-year-old right-handed Caucasian male, who is a resident of a midwestern state of the United States, follows a non-vegetarian diet, was diagnosed with renal cell carcinoma a few years back with no other co-morbidities. He was managed with unilateral nephrectomy a couple of years ago with a baseline creatinine of 1.5 mg/dl. The patient presented to the emergency department for acute onset dramatic decline in mental status. There was no history of a fall or external injury. It started a day before with gait disturbance associated with a decrease in spontaneous limb movements. There was no giddiness, sensory complaint of any kind, or any visual disturbances. His kindred noticed staggering and reduced speech output with decreased spontaneity in response, with a flat emotional status. No similar episode was noticed earlier, nor was there any history of alcohol or illicit drug consumption, although there was a history of non-catarrhine meat intake in the last few days.

Diminished attention with a lack of motivation appeared while examining the patient. On examination, the patient was fully conscious and alert with no focal neurological deficit; cranial nerves were intact, and meningeal signs were absent. There was a decreased spontaneity of speech with a latency in responding; he was able to utter only a few words. Extraocular movements were full with equal-sized pupils reactive to light. The gait was slow, with a waxy rigidity of limbs noted to passive movements. Reflexes were intact. The patient was afebrile with no signs of external injury, dehydration, or any collaborative infective foci findings. Detailed clinical evaluation supported by brain imaging ruled out stroke. Other systemic examinations were unremarkable. MRI brain scan with vascular imaging ruled out potential frontal or temporal hypoperfusion. Awake and sleep electroencephalogram (EEG) further ruled out any ictal or postictal state of convulsion. Related investigation done in this case is illustrated in Table 1 below.

**Table 1:** Relevant investigations including the tick borne disease panel are elaborated in tabular format ESR: erythrocyte sedimentation rate; CT: computerized tomography; MRI: magnetic resonance imaging; EEG: electroencephalogram; CSF: cerebrospinal fluid; ECG: electrocardiogram; IgG: immunoglobulin G; IFA: immunofluorescence assay

Investigation	Result	Reference
Complete blood count with differentials	Normal	-
ESR	16	10-20 mm in first hour
Glucose, random	5.4	5.0-7.8 mmol/ L
Liver function test	Normal	-
Creatinine	1.5 mg/dl (Baseline)	0.7-1.1 mg/dL
Routine urine examination	Normal pH, no proteinuria	-
Comprehensive drug screening test	No abnormality	-
Electrolytes (sodium, potassium, chloride)	Normal range	Sodium 136-146 mEq/L; potassium 3.5-4.5 mEq/L; chloride 96-106 mmol/L
Calcium (ionized)	Normal	4.8-5.6 mg/dL
CT scan brain	No bleed	-
MRI brain with vascular imaging of brain and neck vessels	No ischemic stroke, no demyelination, vessels patent, no hypo-perfusion	-
EEG	Normal background, normal waves in awake and sleep	-
CSF analysis	Proteins were elevated (80 mg/dl), rest all in normal range. Opening and closing pressures were not measured.	-
ECG	Sinus rhythm. No arrthymia, QTc = 430	-
Ehrlichia chaffeensis, IgG antibody (gram-negative Rickettsiales bacteria transmitted by the lone star tick)	1:256 (Positive)	IFA titers > or = 1:64 suggests current or previous infection
Anaplasma phygocytophilum, IgG (gram-negative bacterium transmitted by tick bites)	1:512 (Positive)	IFA titers > or = 1:64 were considered positive
Galactose 1,3 alpha galactose specific IgE antibody (aka anti-α-Gal specific IgE antibody; related to lone star tick bite and non primate mammal meat consumption)	0.54 kU/L (Positive)	Antibody titers>0.35 kU/L are considered elevated

Our clinical impression was abulia as it met its required diagnostic criteria. The cerebrospinal fluid (CSF) analysis found elevated protein at 80 mg/dl, otherwise unremarkable. CSF opening pressure was not measured. The patient was treated with intravenous magnesium sulfate infusion of 500 mg BID dose combined with dextrose 5% for three days which helped with his symptoms. During treatment, the patient was on a cardiac monitor with magnesium levels being monitored frequently. A trial of levodopa/carbidopa was started, however his speech worsened drastically and was discontinued. After mild improvement in one week, the patient was discharged and transferred to a skilled nursing facility for rehabilitation. Here the patient experienced two more spells of apathy with transient aphasia associated with no will to move limbs; each lasted for a couple of hours before spontaneous recovery. Since then, these episodes drastically improved with no residual deficit, and the patient did not opt for an emergency visit. A new onset holocranial mild pressing headache started several times a week lasting for a few hours, and responded to oral paracetamol. Occasionally, there was a component of migrainous throbs associated with moderate fatigue but no nausea or vomiting. There was no associated fever, photophobia, or phonophobia. The patient claimed minimal interference in his routine daily activities because of these headaches. Repeat brain imaging ruled out any bleed or ischemic stroke. Topiramate was started but provided minimal benefit.

The patient’s wife recalled multiple episodes of tick bites recently. Immediately, a detailed tick-born disease panel was ordered. The positive titers of symbiotic organisms carried by these ticks can be seen in Table 1. Anti-α-Gal specific IgE antibody titers were also found to be positive. The patient started a diet void of mammalian meat. Surprisingly, all previous symptoms of fatigue, spells of aphasia, stuttering speech, decreased limb movements, disturbed gait along with headache resolved drastically in a few weeks time with no residual neurological deficit on starting this no-meat diet.

## Discussion

**Figure 1:**
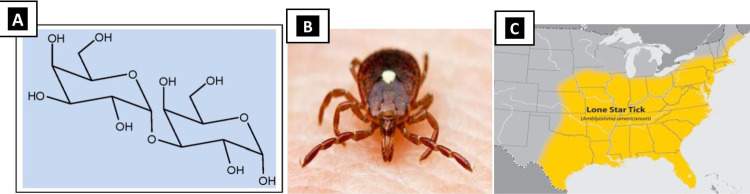
A: structure of α-Gal; B: lone star tick (Amblyomma americanum); C: geographical distribution of lone star ticks in the southeast United States A: structure provided by MK, details in acknowledgments, C: image courtesy: cdc.gov; https://www.cdc.gov/ticks/maps/lone_star_tick.pdf α-Gal: galactose-α-1,3-galactose

Lone star ticks (Amblyomma americanum), identified by a conspicuous white spot on the shield of females, contain Galactose-α-1,3 galactose (a sugar derivative commonly known as α-Gal) in their saliva, which probably comes after a deer blood meal. It is geographically restricted to southeastern regions of the United States, namely Tennessee, North Carolina, Arkansas, Virginia, and Southern Missouri (Figure 1) [2]. Humans do not have α-Gal; our immune system recognizes it as a foreign target and generates necessary antibodies to tackle it once it gets into our system [3,4].

Neoteric testament demonstrates greater and unstable atheromatous plaque load in α-gal sensitized coronary artery disease (CAD); well supported by high titers of IgE immunoglobulin specific to α-gal [3]. The possible neuro-psychiatric burden due to α-gal syndrome (AGS) is unknown. Although the use of α-gal oligosaccharide epitope as a target for antigen-presenting cells (APC) for enhancing vaccines immunogenicity [5] is well known, the same sugar moiety has caused some disastrous neurological disorders in the past, particularly in α-gal sensitized individuals (Table 2) [6,7]. This raises concern that α-Gal could have a possible potential role in striking down neuronal tissues.

**Table 2:** A tabular format illustrating vaccines, possible role of α-Gal as an allergen and the neurological conditions it portrayed GBS: Guillain-Barré syndrome; H1N1: influenza A virus subtype; α-Gal: galactose-α-1,3-galactose; MMR: measles mumps rubella; HLA: human leukocyte antigen; CSF: cerebrospinal fluid

Sl. No.	Vaccines	Adverse effects	Reference
A	Possibility of α-Gal allergy	Neurological complication associated with vaccines	-
1	MMR vaccine (α-Gal allergen, bovine calf serum and gelatin used)	vaccine-associated anaphylaxis	[7]
2	Zoster vaccine 2014 (α-Gal allergen, bovine calf serum and gelatin used)	Vaccine-associated anaphylaxis	[7]
B	Vaccines- no identifiable link	Neurological complication associated with vaccines	-
1	Monovalent H1N1 swine influenza vaccine, 1976	GBS (362 cases were observed 6 weeks post influenza vaccination; about 8.8 fold increase from background incidence)	[8]
2	H1N1 vaccine- Pandemrix (used in Northern Europe, 2009)	Narcolepsy spike in Scandinavia (surfaced after a year of vaccination). Low hypocretin levels in CSF with loss of hypocretin producing neurons within hypothalamus seen with narcolepsy. HLA association further suggests autoimmune nature.	[6]

Hypocretins (aka orexins), peptides synthesized by lateral hypothalamus, are allied with sleep-wakefulness, energy, and neuroendocrine homeostasis [6,9]. Cerebrospinal fluid (CSF) samples obtained from neuro-immunological disease (NID, namely Guillain-Barre syndrome and multiple sclerosis) and non-NID patients (expressly Parkinson’s disease, epilepsy, Alzheimer's disease, stroke) identified Guillain-Barre syndrome (GBS) as the only subset with markedly lower hypocretin-1 (orexin-A) level compared to all [10]. Exceedingly low hypocretin-1 levels in two GBS patients (65 pg/mL and 123 pg/mL, compared to 293± 42 pg/mL level in the non-NID group) clinically analogized well with the severity of illness [10]. Orexin levels are related to GBS pathology, and since GBS and AGS share a common pathomechanism, perhaps there might be a possibility that orexins could inflict a role in the neurological complication of AGS and is a subject for further research.

**Figure 2:**
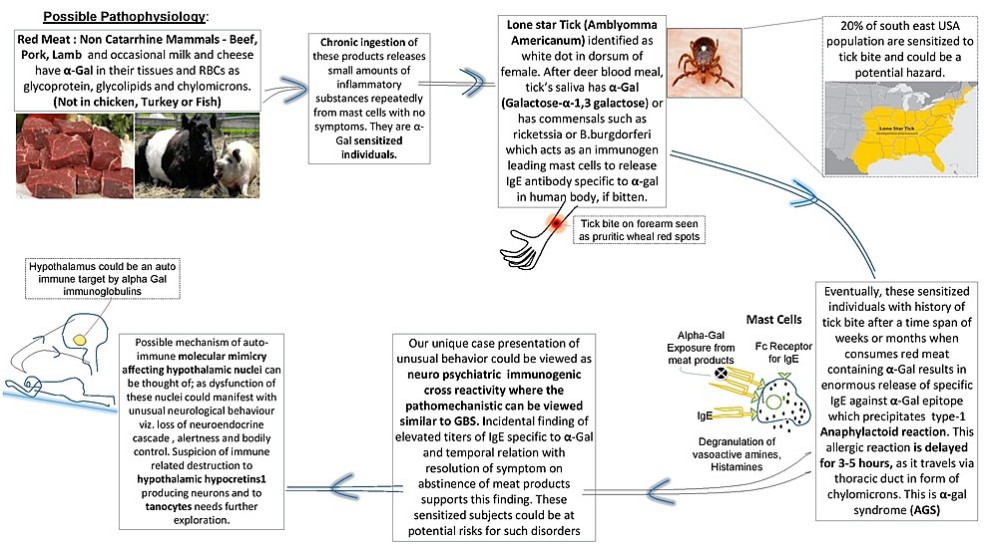
Possible pathomechanisms of α-Gal induced molecular mimicry affecting hypothalamus Consumption of red meat post tick bite eventually causes tick’s saliva-induced IgE immunoglobulin to cross-react with α-gal oligosaccharide epitope found in consumed non-catarrhine meat products. This riposte releases an enormous amount of specific IgE precipitating delayed type-1 anaphylactoid reaction. Activated Fc gamma family receptors on mast cells, macrophages, and basophils can mount IgG response parallel to IgE. Possible pathomechanisms of α-gal induced molecular mimicry, perhaps affecting the hypothalamus has the potential to precipitate abnormal neuro-psychiatric symptoms. α-gal: galactose-α-1,3-galactose; AGS: α-Gal syndrome; IgE: immunoglobulin type E; GBS: Guillain-Barré syndrome Author’s own creation

Recently, Fuente et al. established a common pathophysiology between GBS and alpha-Gal syndrome (AGS) [4]. GBS, a neuro-paralytic immunological autoimmune disease, embraces an oligosaccharide structure in the lipopolysaccharide surface of *Campylobacter jejuni*. This structure kindles molecular mimicry by inducing the yield of anti-ganglioside antibodies (namely GM1, GM2, GQ1b) that cross-react with human gangliosides and glycolipids leading to myelin and axonal destruction mediated through complement and antibody-bound macrophages, shattering the tolerance to self-antigens [4]. A similar pathomechanism where specific IgE antibody generated against a different oligosaccharide, α-Gal, present in tick saliva protein and nonprimate mammal meat affecting vital systems in humans, thus demonstrating an excellent example of anti-α-Gal specific antibody vanquishing immune tolerance (Figure 2) [4].

The lateral hypothalamus, along with arcuate and ventromedial nucleus, receives neurons from tanycytes, glial-like character cells which line the third and fourth ventricles expressing intermediate filament protein, play a role in the exchange of signals between CSF and brain parenchyma along with hypothalamus necessary for modulating important bodily function [9]. Neurons of above hypothalamic nuclei are sensitive to these circulating signals of leptins, grehlin, insulin, and glucose and further relays in regulating wakefulness, feeding, appetite, arousal, energy status, and many more [9]. We believe that perhaps there could be a possible role of the hypothalamus along with tanycytes in the neurological manifestation of AGS.

## Conclusions

Our patient experienced an abnormal neuro-psychiatric behavior after consuming non-catarrhine meat products with an unnoticed tick bite incidence prior. The tick bite disease panel revealed *Ehrlichia chaffeensis* and *Anaplasma phygocytophilum* antibody titers positive. Evidence of anti-α-Gal immunoglobulin titers with a symptomatic temporal resolution paralleled red meat abstinence raises concern of autoimmune molecular mimicry shattering immune tolerance. Abnormal neurological behavior affecting the hypothalamic nucleus (viz. lateral, arcuate, ventromedial) by α-Gal either directly or indirectly through orexins or chemo-sensitive hypothalamic tanycytes is a matter of research and needs further exploration. Similar neurological incidents in the future would strengthen our findings.
